# Evolution of WHO COVID-19 mask guidelines amid intense demands for rapid advice

**DOI:** 10.1371/journal.pgph.0003778

**Published:** 2024-11-08

**Authors:** Nathan Ford, Hannah Hamilton Hurwitz, Roger Chou, Vicky Willet, May Chu, Mitchell J. Schwaber, Kathleen Dunn, João Paulo Toledo, Alice Simniceanu, Madison Moon, Rebekah Thomas, Benedetta Allegranzi, April Baller

**Affiliations:** 1 World Health Organization, Geneva, Switzerland; 2 Pacific Northwest Evidence-based Practice Center and the Department of Medical Informatics and Clinical Epidemiology, Oregon Health & Science University, Portland, Oregon, United States of America; 3 Colorado School of Public Health, Anschutz Medical Campus, Aurora, Colorado, United States of America; 4 National Center for Infection Control, Israel Ministry of Health, Jerusalem, Israel; 5 Faculty of Medical and Health Sciences, Tel Aviv University, Tel Aviv, Israel; 6 Infectious Disease and Vaccination Programs Branch Agency: Public Health Agency Canada, Ottawa, Ontario, Canada; University of Hong Kong Li Ka Shing Faculty of Medicine, HONG KONG

## Abstract

During a health emergency, there is an urgent need to rapidly develop guidelines that meet minimum quality standards, as exemplified by the development of WHO guidelines on mask use in health care and community settings during the COVID-19 pandemic. Between January 2020 and October 2023, WHO developed 21 guideline updates on the use of masks as part of infection prevention and control (IPC) practices. Guideline developers had to deal with an ever-growing volume of evidence of variable quality. Initially, indirect evidence drawn from other severe respiratory illnesses and established minimum requirements for IPC were used. As direct evidence began to emerge, WHO commissioned a living systematic review on mask use in June 2020, which formed the basis of evidence-to-decision making. As more evidence became available, additional considerations were incorporated into the process of recommendation formulation, including harms, acceptability, feasibility and resource use. Target populations for the mask guidelines expanded to include the general public, including children. A broad range of disciplines supported guideline development, including IPC, epidemiology, infectious diseases, occupational health, engineering, pneumology, paediatrics, and water, sanitation and hygiene, as well as civil society representatives. Additional expertise was engaged in the areas of ventilation and aerobiology to expand the range of perspectives regarding modes of transmission. Despite challenges, the experience of rapidly and regularly updating advice on mask use during an emergency health response has shown that it is possible to apply the minimum standards for ensuring the guideline methodology is trustworthy and transparent, with increasing rigor over time as evidence improves. Overall, the experience of developing guidelines during the COVID-19 pandemic underscores the importance of adapting to evolving evidence, incorporating diverse perspectives, and maintaining transparency to ensure a rigorous methodology and in return guideline trustworthiness and effectiveness.

## Introduction

Guidelines are critical in shaping clinical practice and guiding public health and social measures. The guideline development process at the World Health Organization (WHO) follows the highest international quality standards in its guideline development process, to ensure transparency in decision making and formulating evidence-based recommendations [1,2]. During a health emergency response, there is an urgent need to develop guidelines within a compressed timeframe, and a number of key steps may need to be abbreviated or postponed. Nevertheless, interim guidelines need to adhere to minimum quality standards, to ensure that recommendations are supported by the best available evidence and diverse and multidisciplinary perspectives. Minimum standards require steps are taken to: minimize bias; apply transparent processes and explicit, reproducible methods; acknowledge potential limitations; and consider the needs and interests of people affected by the recommendation [3]. Adherence to minimum quality standards is particularly important during emergencies if public health officials are to provide a source of credible and trustworthy advice amid an infodemic of sometimes false or misleading information [4].

The COVID-19 pandemic required WHO to rapidly develop guidelines across a broad range of intervention areas, from the clinical management of individual patients [5] to public health considerations for international travel [6]. Knowledge about the novel causative pathogen evolved swiftly, and recommendations needed to be formulated and updated during this period of rapidly generated scientific evidence of highly variable quality [7,8].

**Fig 1:**
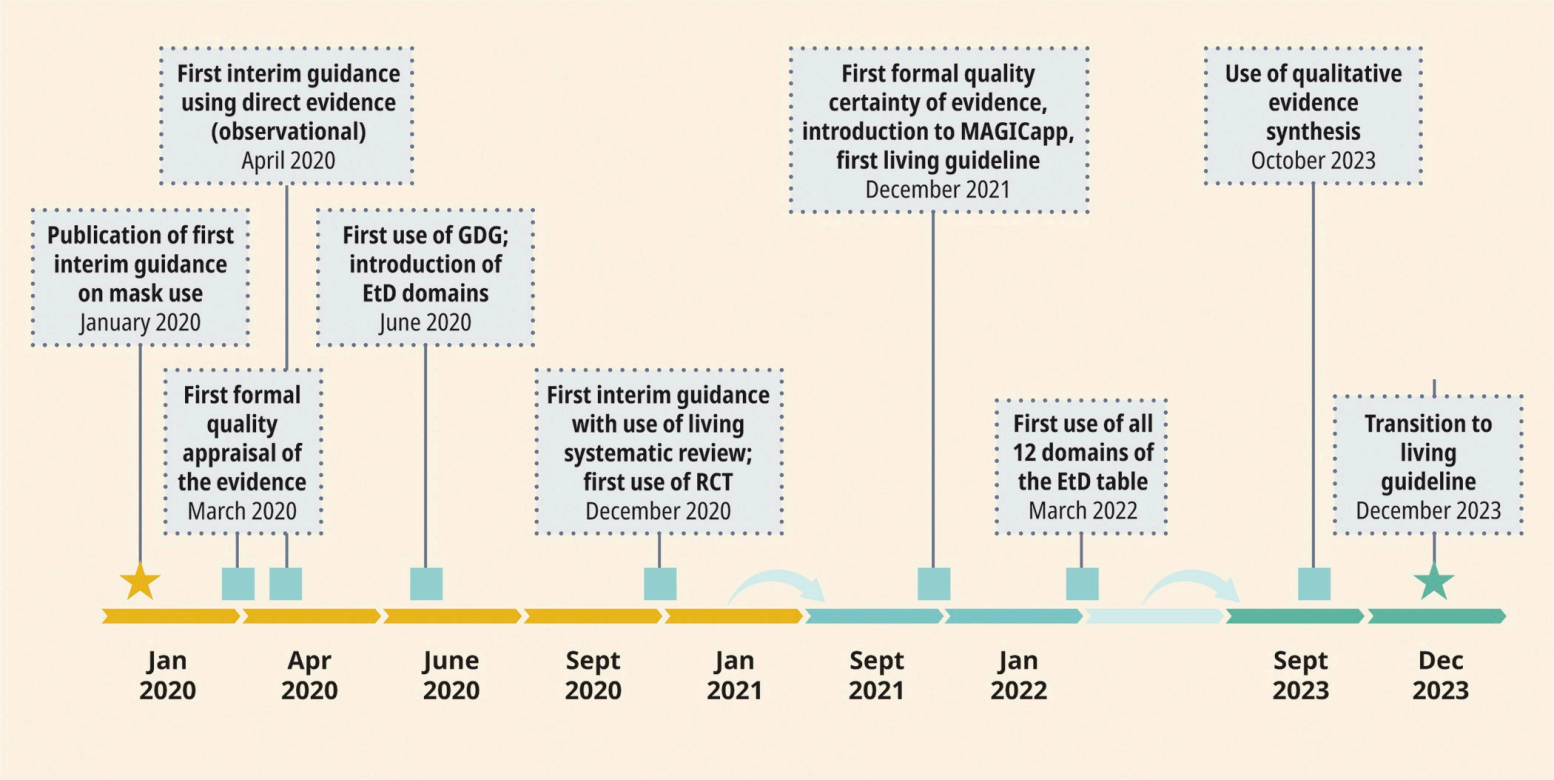
Evolution of WHO COVID-19 Mask Guidance. EtD, Evidence to Decision framework; GDG, Guideline Development Group; RCT, randomized controlled trial.

This article takes the example of mask use in the health care and community settings to describe the progressive application of the minimum standards required for developing trustworthy guidelines [9] during a public health emergency (Fig 1).

### Evolution of the guidelines

Between January 2020 and October 2023, WHO developed 21 guideline updates on the use of masks as part of infection prevention and control (IPC) practices to prevent the spread of severe acute respiratory syndrome coronavirus 2 (SARS-CoV-2). Considering the uncertainty and rapid emergence of new evidence, each WHO guideline noted that recommendations would be updated as soon as new information became available.

Development of guidelines during the acute phase of an emergency required the rapid appraisal of evidence that was emerging from different disciplines; expansion of the evidence-to-decision considerations; expansion of the range of expertise; and maintenance of consistency across different WHO documents. The progressive adoption of WHO minimum standards for guideline development are summarised in Table 1.

**Table 1:** Evolution of WHO IPC mask guidelines for health workers and general public during the COVID-19 pandemic^+^.

	Progressive implementation across domains Date corresponds to when a guideline adopted a change in one of the domains						
Domain	January 2020	March 2020	April 2020	June 2020	August 2020	December 2020	December 2021 –October 2023
Type	Interim guidance	Interim guidance	Interim guidance	Interim guidance	Interim guidance	Interim guidance	Living guideline
Types of evidence	Indirect	Indirect	Indirect & direct	Indirect & direct	Indirect & direct	Indirect & direct	Indirect & direct
Types of direct evidence	None	None	Observational studies	Observational studies	Observational studies and randomized trials	Observational studies and randomized trials	Observational studies and randomized trials
Living systematic review	No	No	No	No	Yes	Yes	Yes
Domains	Benefits	Benefits	Benefits & harms	Benefits & harms Acceptability Resource implications*	Benefits & harms Acceptability Resources implications*	Benefits & harms Acceptability Resources implications*	Benefits & harms Acceptability Resources implications*
Population	Adults	Adults	Adults	Adults	Children	Adults & children	Adults & children
Setting	Home care, health care & community	Home care, health care & community	Home care, health care & community	Home care, health care & community	Community	Home care, health care & community Physical exercise	Home care, health care & community Physical exercise
GDG listed	No	No	No	Yes	Yes	Yes	Yes
ERG listed	No	No	No	Yes	Yes	Yes	Yes
Format	Word document	Word document	Word document	Word document	Word document	Word document	PDF & app

GDG, Guidelines Development Group; ERG, External Review Group.

* Considerations on the use of fabric masks provided for situations with limit access to medical masks.

### Rapid appraisal of emerging evidence

During the initial months of the pandemic, there was a lack of evidence on mask use for preventing SARS-CoV-2 infection. The first two guidelines that included references to mask use, published in January and March 2020, were based on guidance issued during previous emergencies involving other severe respiratory illnesses due to coronaviruses–Middle Eastern Respiratory Syndrome (MERS) [10] and Severe Acute Respiratory Syndrome (SARS) [11]. Initial recommendations were also based on established minimum requirements for IPC, together with a systematic review that had been published in 2011 on physical interventions to reduce the spread of respiratory viruses other than SARS-CoV-2 [12–14].

**Fig 2:**
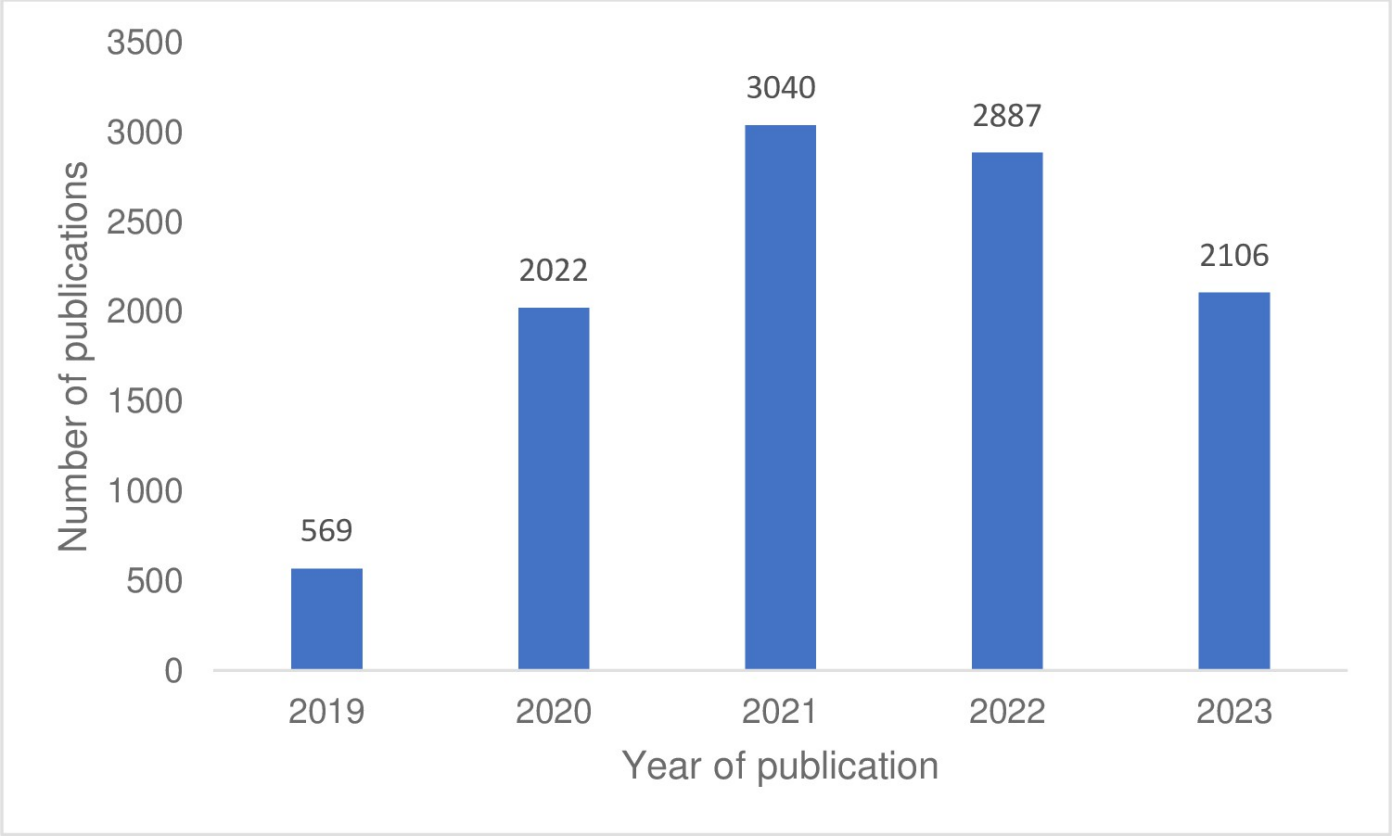
Published studies on mask use from 2019 to 2023. Data from PubMed. Search term “masks[tiab]”.

The evidence-base for mask use to prevent respiratory illnesses grew rapidly. The number of published studies on mask use increased four-fold from 2019 (the year before the pandemic) to 2020 (Fig 2). However, these studies were of highly variable quality, with findings difficult to interpret due to high risk of bias and methodological limitations. Common limitations included potential recall bias and inability to measure or fully control for potential confounders, such as implementation of other measures used to reduce infection and exposure pathways.

To assist with evidence retrieval, WHO established the COVID-19 global literature database in January 2020, which provided a central repository fed by 30 major databases including PubMed, Embase, CINAHL, and GIM (https://search.bvsalud.org/global-literature-on-novel-coronavirus-2019-ncov/) [15]. The very rapid emergence of evidence included a large number of studies published on pre-print servers without peer review. A search of the WHO COVID-19 global literature database found that during 2020, 51 articles with “masks” in the title were published during 2020 on preprint servers (without peer review); three years on, almost a fifth (18%) of those articles remained unpublished, without having undergone any peer review. (The database was archived in January 2024.)

In June 2020, WHO commissioned a living systematic review on the use of masks for the prevention of SARS-CoV-2 infection [16]. This review has been used to inform the guideline updates since December 2020 [17], together with indirect evidence for other respiratory infections. The evidence on SARS-CoV-2 remained mostly observational and related to other respiratory infections (including other coronavirus infections and influenza/influenza-like illness); evidence from randomized trials was available only for influenza/influenza-like illness. Abridged quality assessment tools were applied, with the key limitations (e.g., recall, selection, or participation bias, issues with outcome evaluation, and analytical methods) of each study noted [18,19]. In December 2020, the search strategy was expanded to include ecological data that assessed the effectiveness of universal masking policies in different geographical settings and populations [20].

In 2021, the findings of two large randomized trials of masks for prevention of SARS-CoV-2 infection in community settings became available: an individual randomized trial from Denmark which assessed the protective effect of wearing a mask outside the home [21], and a cluster randomized trial from Bangladesh which investigated mask promotion, published as a pre-print in 2021 and in full in 2022 [22]. A third randomized trial, conducted in hospital settings and published at the end of 2022, compared the protective value of masks with that of respirators worn by health workers [23].

During this period, guideline developers had to deal with a fluctuating evidence landscape, which was marked by an ever-growing volume of evidence of variable quality. Such evidence required rapid and robust appraisal, synthesis and translation to practice, which was provided by the WHO secretariat supported by the methodologist. In later stages of the process, the quality of the studies was evaluated using criteria specific for RCTs and observational studies and the certainty of evidence was determined following the Grading of Recommendations, Assessment, Development and Evaluation (GRADE) process.

### Expanding the range of evidence-to-decision considerations during health emergencies

Guideline recommendations are based on the balance of benefits and harms and other considerations. The first guidelines drew on evidence of protective efficacy based on established practices and evidence from other respiratory pathogens, including recommending the use of respirators during aerosol generation procedures.

As countries began to adopt these recommendations and more evidence became available, WHO guidelines incorporated additional considerations. Harms associated with mask wearing were incorporated as a consideration in June 2020, including potential self-contamination, breathing difficulties, less adherence to other preventive measures, and limited supplies. A systematic review of potential harms (not commissioned by WHO) was published in mid 2020 [24].

Since June 2020, considerations of acceptability, feasibility and resources use were incorporated, drawing on the perspectives of the Guideline Development Group (GDG) and the published literature. A key concern was availability of masks for all frontline health care workers caring for patients with SARS-CoV-2, and others in health facilities, due to global mask shortage, especially during the first year of the pandemic. These guidelines noted that the use of medical masks in the community may divert this critical resource from the health workers and that these should be reserved for health workers and at-risk individuals, with respirators reserved for higher risk situations. The WHO GDG determined that in the context of severe medical mask shortage in health care settings, extended use or reprocessing PPE (using previously worn PPE after decontamination or reprocessing methods) of masks was acceptable, provided that appropriate methods for management and decontamination were implemented [25]. Technical specifications on the use by the general public of alternatives to medical masks were included in these guidelines.

The GRADE approach uses evidence to decision frameworks that includes 12 domains that guideline panels should consider when making a decision. These domains include the priority of the problem desirable and undesirable effects of the intervention; certainty of the evidence; value placed on the outcomes; resource requirements; impact on health equity; and feasibility and acceptability of the intervention [26]. From March 2022, WHO considered all 12 domains within the guidelines and employed the use of the The MAGIC authoring and publication platform (MAGICapp) platform to facilitate the process. MAGICapp [27] is a web-based platform that facilitates the development, updating, and dissemination of digitally structured guidance using the GRADE methodology. At this time, in consideration of the rapid spread of the Omicron variant of concern, WHO provided additional guidance on the settings and conditions where it recommended that respirators be used for care of patients with suspected or confirmed COVID-19 [28].

To improve the generalizability and validity of the guidelines across a range of contexts, WHO commissioned five qualitative evidence syntheses, which were included in the October 2023 guideline. These reviews sought to review the perceptions and experiences of health and care workers of personal protective equipment (including masks), physical barriers, and distancing interventions for the prevention of COVID-19, to better inform the evidence-to-decision making process (e.g., acceptability, feasibility, values and preferences) [29].

To prevent widespread uncertainty and ensure consistent advice across major guideline development agencies such as the US Centers for Disease Control, the WHO secretariat kept in regular contact with these agencies; guidance from these organizations was consistently presented to the GDG throughout the guideline development process. While there were minor variations in these guidelines, these did not determine the GDG’s decision making process which remained independent.

### Expanding the target populations

Initially, guidelines were directed at public health and infection prevention and control professionals, health care managers, and health and care workers. The January 2020 and March 2020 guidelines were intended for health workers and caregivers at home, with limited reference to the general public [30]. However, there was a need to expand the target populations for the mask guidelines, in response to the evolving demands and the pandemic’s effect on different population groups.

The scope of the guidance was expanded to address specific advice for mask use by the general public in June 2020 [31] (initial consideration was given in April, noting limited evidence and the need to prioritize medical masks for health care workers [32]). Subsequently, in December 2020 advice on mask use during physical exercise was also provided [17]. In August 2020, WHO—together with UNICEF—published guidelines on mask use for children that drew on studies evaluating the effectiveness of mask use in children for influenza and other respiratory viruses, and considered evidence on acceptability [33]. In 2022, in light of the impact of the Omicron variant, WHO updated the guidelines for children [34].

### Expanding the range of expertise

The first rapid guidelines, published in January 2020, were based on MERS-CoV guidance, which was developed in consultation with the WHO Health Emergencies Program (WHE) Ad-Hoc Advisory Panel of Infection Prevention and Control (IPC) Experts for Preparedness, Readiness and Response to COVID-19 and other international experts.

In June 2020, a GDG was established and included 33 members from 22 countries, ensuring gender and geographical balance and representation of a range of disciplines, including IPC, epidemiology, infectious diseases, occupational health, engineering, pneumology, paediatrics, and water, sanitation and hygiene, as well as representing patients and the civil society. Recognizing that there was a need to expand the range of perspectives regarding modes of transmission, in August 2020 additional expertise was engaged in the areas of ventilation and aerobiology. The development of recommendations for mask use by children in August 2020 was supported with technical perspectives sought from UNICEF and the International Paediatric Association. In 2022, a specific GDG was created for the updated guideline on mask use for children.

A formal external peer review process was established as part of the guideline development process from August 2020, including content experts in IPC and infectious diseases, and methods experts in evidence-based medicine. Any important comments received after publication of the guidelines were considered as part of subsequent updating.

Declarations of interest of all GDG and external peer reviews were managed according to standard WHO practice [35].

### Ensuring consistency across different normative documents

To ensure consistency in the interpretation of accumulating evidence for SARS-CoV-2, the GDG ensured that methodological support for all guideline discussions came from the same methodologist. During the emergency, WHO published numerous guidelines, together with other supporting documents, including implementation tools and risk communications resources such as questions and answers (Q&A) infographics and videos. A separate clearance body–the Publications Review Committee–was put in place in order to ensure quality and consistency in normative statements across all documents. By March 2023, the Publications Review Committee had reviewed 1374 guidance documents related to COVID-19. Where relevant, these submissions were cross-checked with the latest IPC guidelines to ensure consistency prior to publication.

### Modes of transmission and infection control

During the early phase of the pandemic there was uncertainty over the dominant modes of SARS-CoV-2 transmission. The terms ‘airborne’, ‘airborne transmission’ and ‘aerosol transmission’ were used in different ways by stakeholders in different scientific disciplines, possibly contributing to confusion about modes of transmission [36]. Notwithstanding these uncertainties, guidance from WHO and other agencies issued advice for infection control since January 2020 that included masking, hand hygiene, and ventilation [37].

The lack of consensus around the definition of airborne transmission highlighted the need for better alignment of terms across disciplines, agencies and pathogens. To address this concern WHO convened a global technical consultation which began in November 2021. This consultation brought together experts across a range of disciplines to seek consensus regarding terminology used to describe the transmission through the air. The consultation aimed to achieve consensus in an area where experts had mutually opposed positions regarding the supporting science.

Consensus was reached on the use of the term ‘infectious respiratory particles’ to move away from a strict dichotomy of particle sizes, and the use of the phrase ‘transmission through the air’ as an umbrella term to describe the transmission via either airborne transmission or direct deposition. Further inter–disciplinary research is needed to build robust evidence regarding transmission mechanisms and infection prevention measures and strategies; such research should include animal models, human challenge experiments, as well as other observational and interventional study designs [36].

## Conclusions

Prior to the pandemic, WHO published guidance on developing rapid advice guidelines in the context of a public health emergency, highlighting the core principles and emphasizing the need for transparent processes with explicit methods [3].

The development of WHO IPC guidelines for mask use during the COVID-19 pandemic involved the adoption of such an approach and the progressive move towards the use of GRADE-based methods for standard guidelines development. A number of key lessons can be drawn from this experience: the establishment of living evidence reviews and living guidelines [38] was critical to streamlining evidence appraisal and supporting guideline panel judgements, with rapid updating of guidelines as soon as new evidence emerged. Inclusion of a wide range of disciplines within the GDG, as well as civil society representatives, was important to ensuring that the guideline incorporated input from a broad range of perspectives, including those provided by end users of the guidelines. The guideline development process highlighted the need to develop a system to quickly assemble a group of advisors from diverse backgrounds for formulating recommendations during an emergency. Finally, there is a need to establish robust monitoring and evaluating systems for emergency guidelines, with mechanisms for real-time feedback to improve and clarify guidelines.

Initially, the guidance had a limited focus and were informed by indirect evidence of very low certainty using abridged processes to allow for very rapid development. As the results of studies using robust methodologies became available, guideline development transitioned to relying on improved evidence and more standard processes. Nevertheless, the majority of studies were observational with important methodological limitations, and most of the recommendations that were developed were based on low or very low certainty evidence. Recommendations on the use of masks in the community and the choice of medical masks versus respirators for health care workers were initially informed by very low certainty evidence but later included the results of randomized trials [21–23]. An important part of securing guideline trustworthiness is ensuring that recommendations put forward in guidelines are in line with the certainty of the underlying evidence. The extent to which health providers and the general public trust the advice that is issued by normative bodies is a major determinant of uptake and acceptance [39].

In 2020 WHO published an R&D Blueprint for IPC in the context of the COVID-19 pandemic to identify areas that would benefit from further research. This document noted the following priorities regarding mask use: randomized trials and observational studies to assess the comparative effectiveness of non-medical masks for the general public as well as medical masks and respirators for health care workers [40]—a question already highlighted in 2014 [11]. Notwithstanding the large quantity of scientific publications and associated systematic reviews on mask use for infection prevention and control, the number of high-quality studies involving sufficient numbers of individuals to produce reliable results and using robust study designs to minimize bias, remains extremely limited. The conduct of large, randomized, controlled trials is challenging in the field of IPC, in particular during emergencies, and observational data will continue to be informative. There remains a need for collaborative efforts to improve the knowledge on measures to reduce respiratory infections, including the preventive efficacy of different mask types. Collaboration across multiple disciplines has also been essential to gaining a fuller understanding of modes of transmission of respiratory infections, including SARS-Cov-2.

Despite challenges (Box 1), the experience of rapidly and regularly updating advice on mask use during an emergency health response has shown that it is possible to apply the minimum standards needed for ensuring that the guidelines are trustworthy and transparent, with increasing rigor over time as evidence improves. The experience of developing guidelines during the COVID-19 pandemic underscores the importance of adapting to evolving evidence, incorporating diverse perspectives, and maintaining transparency to ensure guideline trustworthiness and effectiveness. WHO officials are reflecting on the lessons learned during the COVID-19 pandemic so that they will be ready to accelerate the development of much-needed guidelines during any future emergency situations, while ensuring that minimum standards are upheld.

**Box 1. Key challenges**

1. **Rapid Appraisal of Emerging Evidence:** The initial months of the pandemic presented a challenge due to a lack of high-quality evidence on mask use for preventing SARS-CoV-2 infection. The rapid emergence of evidence made synthesizing and interpreting findings challenging.
2. **Variable study quality:** Despite the large quantity of scientific publications on mask use, the number of high-quality studies with robust methodologies remained limited often resulting in low to very low quality of evidence when appraised. Conducting large, randomized controlled trials in the field of infection prevention and control, particularly during emergencies, presents logistical and ethical challenges.
3. **Expanding Evidence Considerations:** There is a need to consider not only the benefits of mask use but also potential harms, feasibility, resource use, and acceptability. Incorporating these expanded considerations required the incorporation of diverse evidence and perspectives, which presented logistical and organizational challenges.
4. **Ensuring Consistency across normative documents:** Ensuring consistency across over a thousand guidance documents was a challenge requiring significant coordination and oversight, including the establishment of new clearance procedures
